# Genome wide association analysis on semen volume and milk yield using different strategies of imputation to whole genome sequence in French dairy goats

**DOI:** 10.1186/s12863-020-0826-9

**Published:** 2020-02-21

**Authors:** Estelle Talouarn, Philippe Bardou, Isabelle Palhière, Claire Oget, Virginie Clément, The VarGoats Consortium, Gwenola Tosser-Klopp, Rachel Rupp, Christèle Robert-Granié

**Affiliations:** 1 GenPhySE, Université de Toulouse, INRAE, ENVT, F-31326 Castanet Tolosan, France; 2 Sigenae, INRAE, 31326 Castanet-Tolosan, France; 3 Institut de l’Elevage, 31326 Castanet-Tolosan, France

**Keywords:** Sequence data, Imputation, Semen, Milk yield, GWAS analysis, French Alpine and Saanen, Goats

## Abstract

**Background:**

Goats were domesticated 10,500 years ago to supply humans with useful resources. Since then, specialized breeds that are adapted to their local environment have been developed and display specific genetic profiles. The VarGoats project is a 1000 genomes resequencing program designed to cover the genetic diversity of the *Capra* genus. In this study, our main objective was to assess the use of sequence data to detect genomic regions associated with traits of interest in French Alpine and Saanen breeds.

**Results:**

Direct imputation from the GoatSNP50 BeadChip genotypes to sequence level was investigated in these breeds using FImpute and different reference panels: within-breed, all *Capra hircus* sequenced individuals, European goats and French mainland goats. The best results were obtained with the French goat panel with allele and genotype concordance rates reaching 0.86 and 0.75 in the Alpine and 0.86 and 0.73 in the Saanen breed respectively. Mean correlations tended to be low in both breeds due to the high proportion of variants with low frequencies.

For association analysis, imputation was performed using FImpute for 1129 French Alpine and Saanen males using within-breed and French panels on 23,338,436 filtered variants. The association results of both imputation scenarios were then compared. In Saanen goats, a large region on chromosome 19 was significantly linked to semen volume and milk yield in both scenarios. Significant variants for milk yield were annotated for 91 genes on chromosome 19 in Saanen goats. For semen volume, the annotated genes include YBOX2 which is related to azoospermia or oligospermia in other species. New signals for milk yield were detected on chromosome 2 in Alpine goats and on chromosome 5 in Saanen goats when using a multi-breed panel.

**Conclusion:**

Even with very small reference populations, an acceptable imputation quality can be achieved in French dairy goats. GWAS on imputed sequences confirmed the existence of QTLs and identified new regions of interest in dairy goats. Adding identified candidates to a genotyping array and sequencing more individuals might corroborate the involvement of identified regions while removing potential imputation errors.

## Background

The recent decrease in sequencing costs has made it possible to sequence large numbers of individuals in key livestock species. The VarGoats resequencing program is the logical next step following the ADAPTmap initiative on 50 k genotyping data [1]. The program plans to sequence over 1000 animals of the *Capra* genus from 65 breeds including 44 French Alpine and 37 French Saanen animals. The sequenced individuals include widely used AI bucks and and enabled to perform a preliminary analysis of imputation for subsequent association analyses. They cover an appreciable part of the effective population sizes of both breeds, estimated to be 115 and 98 in Alpine and Saanen respectively (Carillier, 2015, INRAE, personal communication).

Imputation is a more cost-effective method for obtaining a large amount of sequence data for subsequent analysis. A high-density genotyped reference panel is used to predict high-density genotypes in a low-density genotyped population. When possible, imputation to a whole-genome sequence is performed in a stepwise manner starting with the lowest density panel, before moving on to a medium density chip, then a high-density chip and finally imputation to sequence level. In dairy cattle and sheep, this method has proved more efficient than direct imputation from lowest density to sequence [2, 3]. In goats, the only genotyping tool available is a 50 k-chip (Illumina GoatSNP50 BeadChip) [4]. This means that imputation must be carried out directly from 50 k to sequence level.

Genome-wide association studies (GWAS) are commonly used to unravel the genetic architecture of complex traits. The GoatSNP50 BeadChip has led to the detection of a few Quantitative Trait Loci (QTL) regions for milk and type traits in French Alpine and Saanen breeds [5–8]. One causal mutation has been identified [5] and a large zone on chromosome 19 needs to be refined in Saanen goats given the width of the confidence interval, the multiplicity of traits associated with the same region of the chromosome and as no straightforward functional candidate gene was identified [6]. The use of sequence data, rather than chip data, for fine QTL mapping has proved more accurate in various species, such as cattle [9, 10] and poultry [11]. Indeed, chip data consist of only a few variants selected based on their quality (length of the contig, proximity to other SNPs, exclusion of tri-allelic and A/T or C/G SNPs, estimated quality of the probe etc. …), spacing and MAF, therefore variants with low MAF are under-represented. However, rare variants could actually have a significant impact on the phenotypes studied as they might have appeared only recently in the target populations. Sequence data include various MAF profiles and should contain the causal mutations that affect the traits of interest. It is therefore preferable to perform association analyses on the whole-genome sequence (WGS) rather than on chip data that mainly rely on linkage disequilibrium with the nearby causal mutation. Besides, chip data may only contain SNPs (single nucleotide polymorphisms) whereas sequence data include both SNPs and small indels (insertion/deletion).

Few association studies have been conducted on semen production traits in goats. For example, Nickbin et al. [12] investigated the HSP70 gene in Boer goats and Mohammed et al. [13] calculated genetic parameters for the Damascus breed. However, the association of semen production traits to regions of the genome has yet to be investigated in Alpine and Saanen breeds despite their economic importance in the French dairy industry. In France, where around 70,000 artificial inseminations are performed every year with Alpine and Saanen bucks, semen production traits are of major interest. Bucks culled for semen defects represent a burden for the French breeding organization, CapGenes. Indeed, nearly 46% of the 120 to 130 young bucks that enter the progeny testing process are discarded due to semen quality issues.

This study is the first to investigate imputation in dairy goats. Our main objectives were to evaluate the quality of imputation, define the best imputation scenario and finally assess the usefulness of imputed sequence data to identify genome regions associated with semen production and milk yield traits in French Alpine and Saanen breeds.

## Methods

### Data available

No animal experiments were necessary for this study, therefore no ethics committee approval was required. Sequence data were obtained from the VarGoats project using Illumina HiSeq or Illumina NovaSeq technologies, the first step towards a 1000 goat genome project (http://www.goatgenome.org/vargoats.html). The current data bank comprises 808 individuals from *Capra hircus* of various breeds and geographical origins, as well as 21 wild goat individuals. Forty-four French Alpine and thirty-seven French Saanen individuals were sequenced at Genoscope (Evry, France) with an average coverage of 12X. The individuals sequenced were selected to best represent the genetic structure of the current French population: AI bucks from the largest families, maximized haplotype coverage from the population by picking unrelated individuals following the approach described by Druet et al. [14]. However for some research purposes (milk flow trait, specific casein profiles), a few closely related individuals were sequenced and added to the overall dataset. Thus, sequence data include 13 pairs of cousins (*G*_*jk*_ > 0.12) and 7 parent/descendant pairs (*G*_*jk*_ > 0.40). All selected animals except 3 had previously been genotyped using the Illumina GoatSNP50 BeadChip.

A total of 2455 French Alpine individuals (994 males, 1461 females) and 1570 French Saanen individuals (757 males, 813 females) genotyped using the Illumina GoatSNP50 BeadChip were available for imputation. Pedigree information was available and used as the genotyped individuals were closely related to the reference panel of sequences. Data were cleaned using an in-house pipeline as described in Martin et al. (2018). In brief, all individuals with a call rate below 95% or showing pedigree inconsistency (i.e. having more than 10% parent/offspring conflicting SNPs) were discarded. SNP quality control was based on the following inclusion criteria: call rate above 99%, MAF above 1% and Hardy-Weinberg *P*-value above 10^− 6^. After editing, a total of 47,147 synthesized SNPs (out of a total of 53,347) remained on goat autosomes CHI 1 to CHI 29 and were used for subsequent analyses. Marker orders and positions were based on the ARS1 caprine Assembly [15] . The GoatSNP50 BeadChip SNP positions were updated on ARS1 genome assembly as described on the VarGoats website (http://www.goatgenome.org/projects.html) and made publically available by the International Goat Genome Consortium.

### Sequence data quality check and imputation

The sequenced reads were aligned to the goat reference genome assembly ARS1 (https://www.ncbi.nlm.nih.gov/assembly/GCF_001704415.1/) using the Burrows–Wheeler Alignment tool (BWA-MEM version 0.7.15) with default parameters [16].

According to GATK best practices, BAM files were preprocessed: duplicates were removed, indels realigned and base quality score recalibrated with Picard tools version 2.1.1 and Genome Analysis Toolkit (GATK) version 3.7–0 [17]. Variant calling was performed for all GVCF files using GATK HaplotypeCaller and variants were annotated using SnpEff (version 4.3 t) [18] and the NCBI *Capra hircus* annotation release 102 (ftp://ftp.ncbi.nlm.nih.gov/genomes/all/GCF/001/704/415/GCF_001704415.1_ARS1/).

**Fig. 1:**
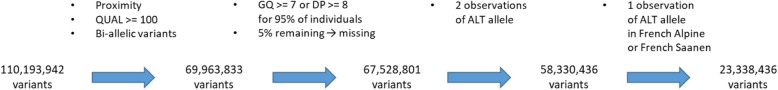
Filtering process of sequence data from 829 individuals (VarGoats 1000 genomes project) including 33 French Saanen and 40 French Alpine goats. QUAL: quality estimated by GATK. GQ: individual genotype quality. DP: individual depth. ALT: alternative allele

Variant calling on the 829 individuals led to the identification of 110,193,942 variants on the 29 autosomal chromosomes: 97,889,899 SNPs and 12,304,043 indels. Among the 829 sequenced individuals, 16 had a mean coverage below 5 (4 French Alpine and 4 French Saanen) and were removed from the data set for subsequent analyses. The sequence dataset therefore consisted of 815 sequenced individuals including 40 French Alpine goats (36 males and 4 females) and 33 French Saanen goats (31 males and 2 females). Quality checks were applied to the sequence variants using the indicators listed in Fig. 1. The thresholds for individual genotype quality (GQ) and individual depth (DP) were set to 8 and 7 respectively by comparing the genotypes of the GoatSNP50 BeadChip SNPs with the sequence variants. The mean GQ and mean DP were 6.7 (± 2.8) and 7.6 (± 4.8) respectively for mismatching SNPs compared with 48.0 (± 16.7) and 11.7 (± 5.1) respectively for the matching genotypes.

After the quality check, only variants with at least one observation of the alternative allele (ALT) in French Alpine or Saanen animals were retained in order to reduce computation time in subsequent analyses and only keep variants of interest in our breeds. Thus, 23,338,436 variants, including 40,491 GoatSNP50 BeadChip SNPs, were kept for imputation. Concordance with the 50 k genotypes was checked. After variant filtering, the individual mean concordance rate was 98.24% (± 1.12) and ranged from 94.00 to 99.96%.

### Imputation of missing genotypes in the sequence reference panel

It should be emphasized that missing genotypes represented on average 4.63% of all the sequence variants for an individual of French Alpine and Saanen breeds. This percentage could attain 66% if sequencing was of low coverage. A within-breed imputation was therefore applied to fill in the gaps. Using a combination of AlphaImpute (v 1.9) [19] and FImpute (v 3.0) [20] gave higher concordance rates than using solely one software while minimizing computation time. We hence imputed filtered sequences using AlphaImpute and FImpute consecutively for French Alpine and Saanen breeds separately. The mean concordance rate between 50 k genotypes and sequence data was 98.62% (± 1.19) after imputation and no missing genotypes remained. For subsequent analyses, as chip genotypes are more reliable than low-depth sequencing, and to avoid spreading genotyping errors down the pedigree, the 50 k markers in the sequencing data were systematically replaced by information from 50 k genotypes, when available.

### Animal phenotypes

#### Male traits

Three semen production traits were recorded on artificial insemination (AI) bucks (Table 1): semen volume in mL (SV), semen concentration in billions of spermatozoa per mL (SC) and number of spermatozoa in billions of spermatozoa (SN). Semen production and quality were analyzed in 305,840 ejaculates from 2865 AI bucks from the CapGenes breeding organization (Mignaloux-Beauvoir, France). Mean yield deviations (YD) per buck were computed from repeated performances (1 to 447 repetitions per buck) then corrected for environmental effects: age, month and year of semen collection, and time between two consecutive samples.

**Table 1:** Available phenotypes for association analysis. Semen production traits included spermatozoa number, semen concentration and semen volume

	Alpine	Saanen
**Milk yield trait (DYD of AI bucks)**	631	483
**Semen production traits (YD of AI bucks)**	668	515

#### Female traits

Milk yield (MY) in kg was also measured as detailed by Martin et al. [5, 6] and analyzed. Daughter Yield Deviations (DYD) were computed for males with at least 10 daughters with records (Table 1). DYDs were the average daughters’ performances corrected for environmental effects and merit of the dam.

### Imputation scenarios of 50 k genotypes to sequence level and quality assessment

Imputation of 50 k genotypes to sequence level was performed using FImpute software (v 3.0) which takes pedigree information into account [20]. The accuracy and efficiency of FImpute, compared with various other imputation tools, has been confirmed [21–23]. Imputation quality was checked before imputing the available 50 k genotypes to sequence level. A leave-one-out scenario was applied to 4 sequenced daughters of 2 different sequenced sires (2 Alpine and 2 Saanen) to maximize the kinship with the reference population. One of the daughters was in turn masked down to a 50 k-equivalent and then imputed. The allele and genotype concordance rates (CR) and Pearson correlations (R) of the true and imputed sequences were then calculated per variant and per MAF profile.

**Fig. 2:**
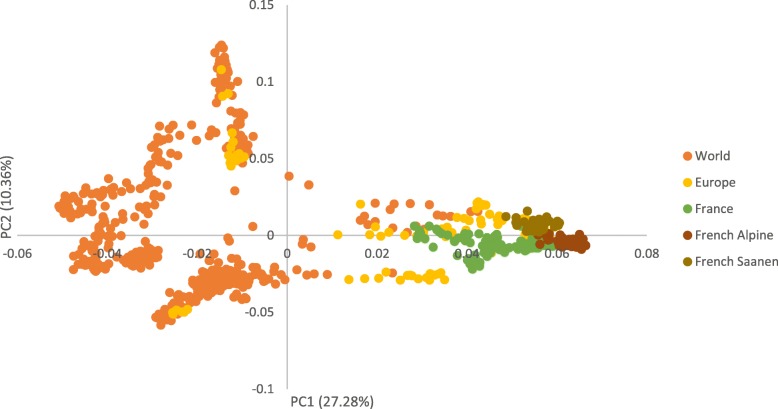
Principal Component Analysis performed on 1,546,528 filtered variants on chromosome 1 using Plink software. The ‘World’ group comprises all sequences available except for wild animals. The ‘Europe’ group comprises all sequences from dairy goats from European countries (therefore excluding Boer, Angora and Creole breeds). The ‘France’ group comprises all sequences of goats from France (excluding Angora and Creole breeds)

Various reference populations were tested based on their proximity to French Alpine and Saanen goats. To accurately build the different reference panels, a Principal Component Analysis (PCA) was performed on chromosome 1 filtered data using PLINK software [24]. Groups were then formed based on the origin of an individual and on the PCA results (Fig. 2). The number of individuals per group is given in Table 2. All sequenced wild individuals were removed from reference populations as they are genetically different from the *Capra hircus* species. We also tried to impute sequences without pedigree while using all sequenced French goats available (excluding Angora and Creole breeds).

**Table 2:** Composition of the different reference populations used for imputation. Details of breed composition available on: http://www.goatgenome.org/vargoats.html

	Number of individuals	
	Alpine	Saanen
Within-breed	39	32
World	793	
Europe	243	
France	169	

### Association analysis

The imputed sequences were subjected to single-trait association analysis for milk and semen production traits using mixed linear models with the *mlma* option of GCTA software [25] and the following model:

$$ \boldsymbol{y}=1\mu +\boldsymbol{x}b+\boldsymbol{u}+\boldsymbol{e} $$

where **y** represents pre-adjusted phenotypes of the trait; μ is the overall mean; b is the additive fixed effect of the variant tested; ***x*** is the vector of imputed genotypes coded in 0, 1, 2 (copy number of the alternative allele); ***u*** is the vector of random additive polygenic effects, ***u***~*N*(**0**, ***G****σ*^2^) with **G** the genomic relationship matrix; ***e*** is the vector of random residual effects normally distributed. The genomic relationship **G** matrix was calculated on 50 k genotypes using PLINK [24].

The four traits were subjected to within-breed association analysis (Table 1). Variants with a within-breed MAF lower than 1% were excluded, leaving 11,933,965 and 12,449,740 variants in Alpine and Saanen goats, respectively, when sequences were imputed within-breed, and 14,695,413 and 15,404,361 variants in Alpine and Saanen goats, respectively, when imputation was performed using the French multi-breed panel. A Bonferroni correction was applied to the significance thresholds to account for multiple testing. The average chromosomal significance level was calculated as follows: −*log*_10_(0.05/(*number of variants*/29)).

The results of the sequence data association analysis were then compared with 50 k-genotypes results, by performing a GWAS on the 40,491 SNPs found both in the filtered sequence data and the cleaned GoatSNP50 BeadChip SNPs.

Annotations were extracted from VCF files for variants with a -log_10_(*p*-value) above the chromosomal threshold. The RumimiR database (http://rumimir.sigenae.org/) [26] was also checked for miRNAs located close to a significant variant.

## Results

### Imputation accuracy

**Fig. 3:**
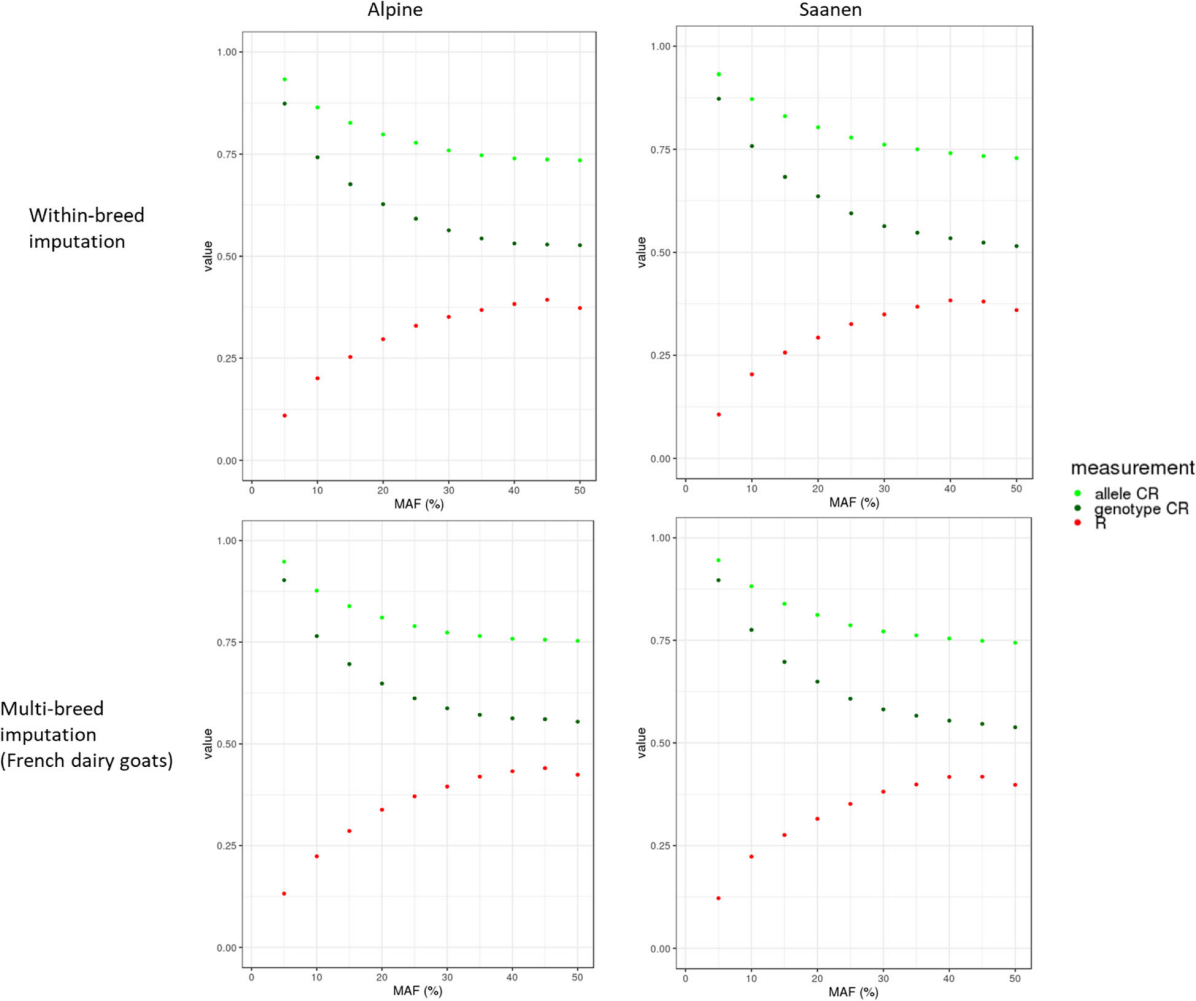
Precision of imputation to sequence level per MAF for within-breed and multi-breed analysis. The precision of imputation was defined as genotype and allele concordance rates (CR) as well as correlation

**Fig. 4:**
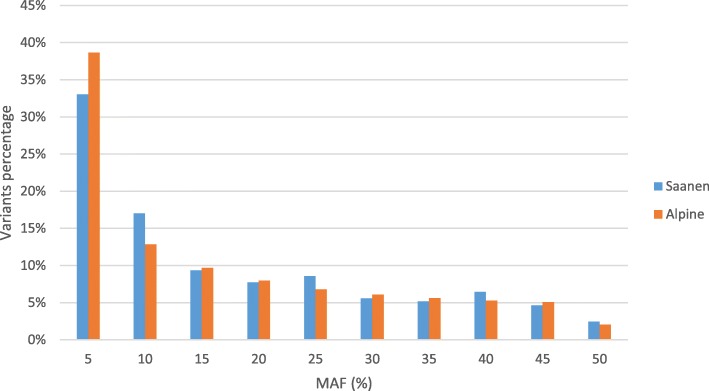
MAF distribution after imputation of the 23,338,436 sequence variants retained after filtering in Alpine and Saanen goats

Allele and genotype concordance rates (CR) and correlations (R) between true and imputed sequences were computed for all chromosomes separately. The mean allele CR, genotype CR and R were calculated per variant and per group of variants with the same MAF. Results per MAF are shown in Fig. 3 for within-breed imputation and imputation with all sequenced French goats. As shown in Fig. 3, imputation using a French multi-breed panel performs slightly better than with a breed-specific reference panel for a specific MAF, regardless of the breed. However, when considering the overall results (Table 3), the difference between the two imputation scenarios is less obvious than when comparing a MAF profile. The high proportion of low MAF in our data tended to flatten the differences as imputation quality measurements are similar in both scenarios for low MAF (from 34 to 38% depending on the breed, Fig. 4). In Saanen, the European multi-breed panel performed better. However, the difference with the French multi-breed imputation is minimal and the computation time increased with the number of sequenced individuals. We therefore chose the less time-consuming multi-breed scenario for further comparisons with within-breed imputation.

**Table 3:** Correlation (R) and concordance rates (CR) between imputed and true genotypes for Alpine and Saanen breeds using different reference populations

Reference population	Pedigree	Alpine			Saanen		
		R	genotype CR	allele CR	R	genotype CR	allele CR
Within-breed	Yes	0.264	0.755	0.867	0.239	0.741	0.859
World	Yes	0.232	0.723	0.850	0.226	0.714	0.845
Europe	Yes	0.264	0.747	0.863	0.251	0.733	0.856
France	Yes	0.265	0.749	0.864	0.248	0.734	0.856
	No	0.211	0.734	0.856	0.198	0.719	0.847

### GWAS analysis

#### Milk yield

**Fig. 5:**
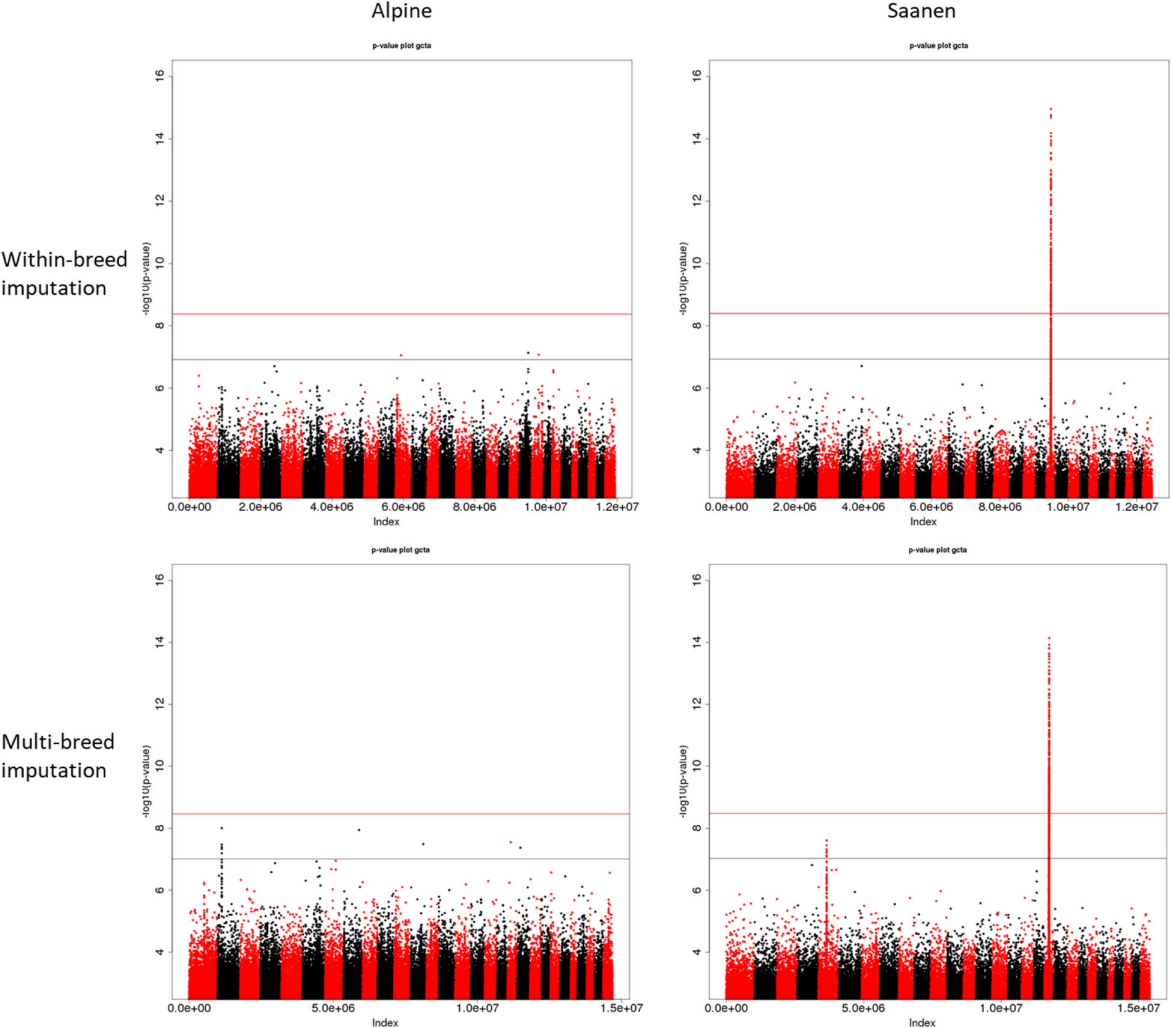
Manhattan plots of genome-wide association studies (GWAS) for milk yield performed on 631 Alpine and 490 Saanen AI bucks for within-breed analysis after within-breed and multi-breed imputation. Chromosome significance threshold in black; genome-wide significance threshold in red

In the Alpine breed, when sequence data was imputed using solely data from French Alpine, only 3 variants out of 11,933,965 reached the chromosomal significance level (*p* − *value* ≤ 1.22 ∗ 10^−7^) for milk yield (Table 4) and no clear signal was detected (Fig. 5). When imputing all 50 k genotypes to sequence level using all sequenced French goats (multi-breed), 9 sequence variants out of 14,695,413 reached the chromosomal significance level (*p* − *value* ≤ 9.87 ∗ 10^−8^) (Table 4) and a clear signal appeared on chromosome 2 between 28.87 and 28.89 Mb (Fig. 5).

**Table 4:** Number of significant variants identified at the chromosome significance level in a population of 483 Saanen and 629 Alpine individuals for both imputation scenarios

	Alpine				Saanen			
Imputation	Within-breed^1^		French goats^2^		Within-breed^3^		French goats^4^	
	sequence	50 k	sequence	50 k	sequence	50 k	sequence	50 k
**milk yield**	3	0	9	0	313	14	448	12
**number of spermatozoa**	0	0	1	0	5	1	51	1
**semen concentration**	2	0	2	0	2	0	8	0
**semen volume**	2	2	2	0	415	11	981	9

Bonferroni thresholds: ^1^ *p* − *value* ≤ 1.22 ∗ 10^−7 2^ *p* − *value* ≤ 9.87 ∗ 10^−8 3^ *p* − *value* ≤ 1.17 ∗ 10^−7 4^ *p* − *value* ≤ 9.41 ∗ 10^−8^

In the Saanen breed, when imputing available 50 k genotypes using only data from sequenced French Saanen, 313 variants out of 12,449,740 reached the chromosome significance level (*p* − *value* ≤ 1.17 ∗ 10^−7^) for milk yield (Table 4), all of which were situated on chromosome 19 between 23.55 and 27.68 Mb. When using a French multi-breed imputation reference panel, 448 variants out of 15,404,361 reached the chromosomal significance level (*p* − *value* ≤ 9.41 ∗ 10^−8^) (Table 4) including 441 on chromosome 19 between 24.70 and 28.15 Mb and 7 on chromosome 5 between 44.80 and 44.81 Mb (Fig. 5).

#### Semen production

**Fig. 6:**
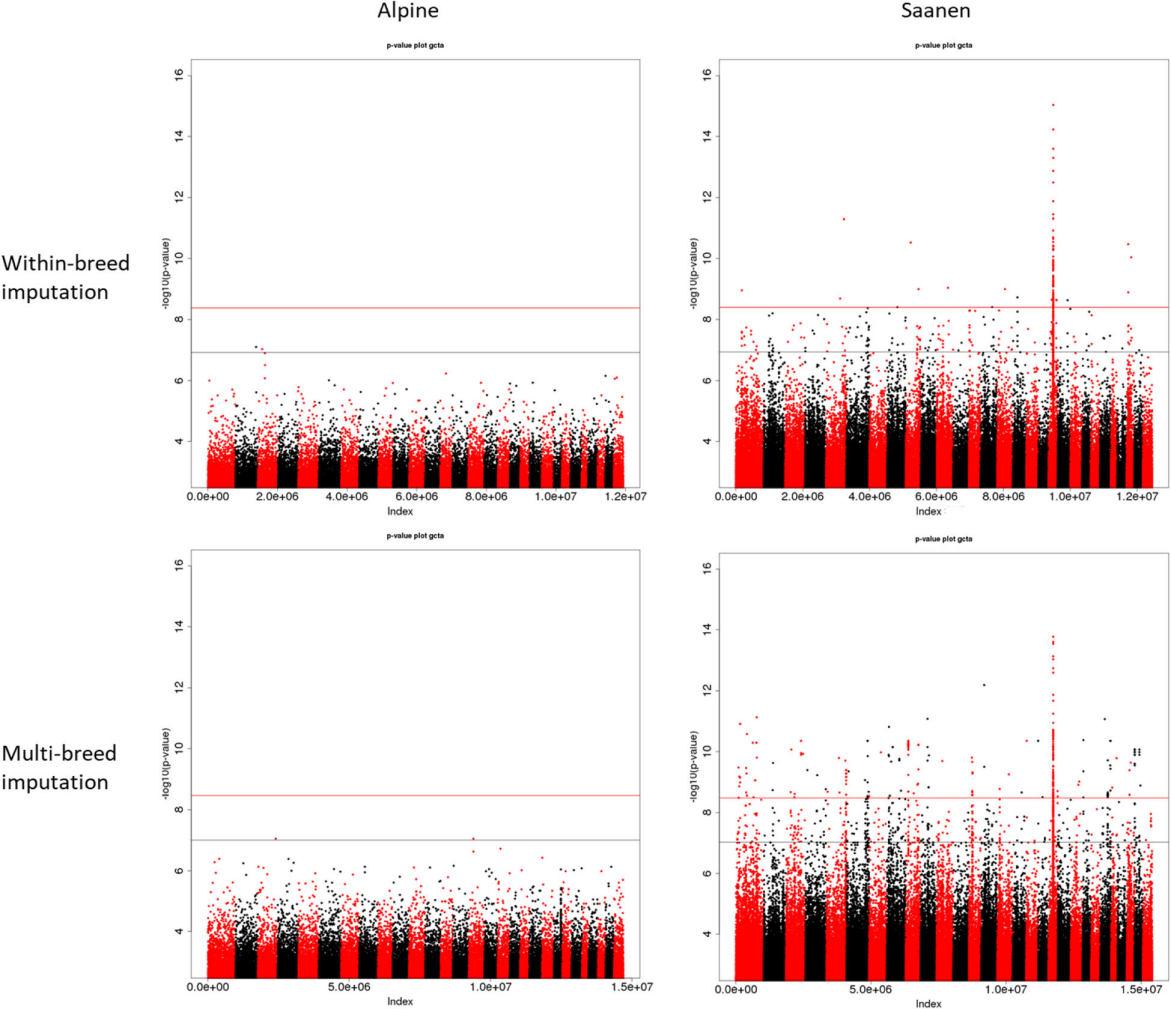
Manhattan plots of genome-wide association studies (GWAS) for semen volume performed on 631 Alpine and 490 Saanen AI bucks for within-breed analysis after within-breed and multi-breed imputation. Chromosome significance threshold in black; genome-wide significance threshold in red

In the Alpine breed, no clear signal was observed for semen production traits with sequences imputed either from sequenced Alpine individuals or the French goat panel (Fig. 6).

In the Saanen breed, a wide significant signal was found on chromosome 19 using within-breed imputation, spanning a region from 24.5 to 27 Mb. The signal was most significant for semen volume for which 209 variants reached the chromosome significance level (Table 4). However, 206 other variants were found to show significant association with this trait on the rest of the genome. When imputing the available 50 k genotypes in Saanen individuals using French goat sequences, 981 variants reached the significance level for semen volume genome-wide but only 23.8% were found on chromosome 19 (Fig. 6). A small signal was also observed for SN on chromosome 19 when using a multi-breed panel, however out the 51 genome-wide significant variants (Table 4) only 4 were located on chromosome 19.

#### Comparison with 50 k genotypes

**Fig. 7:**
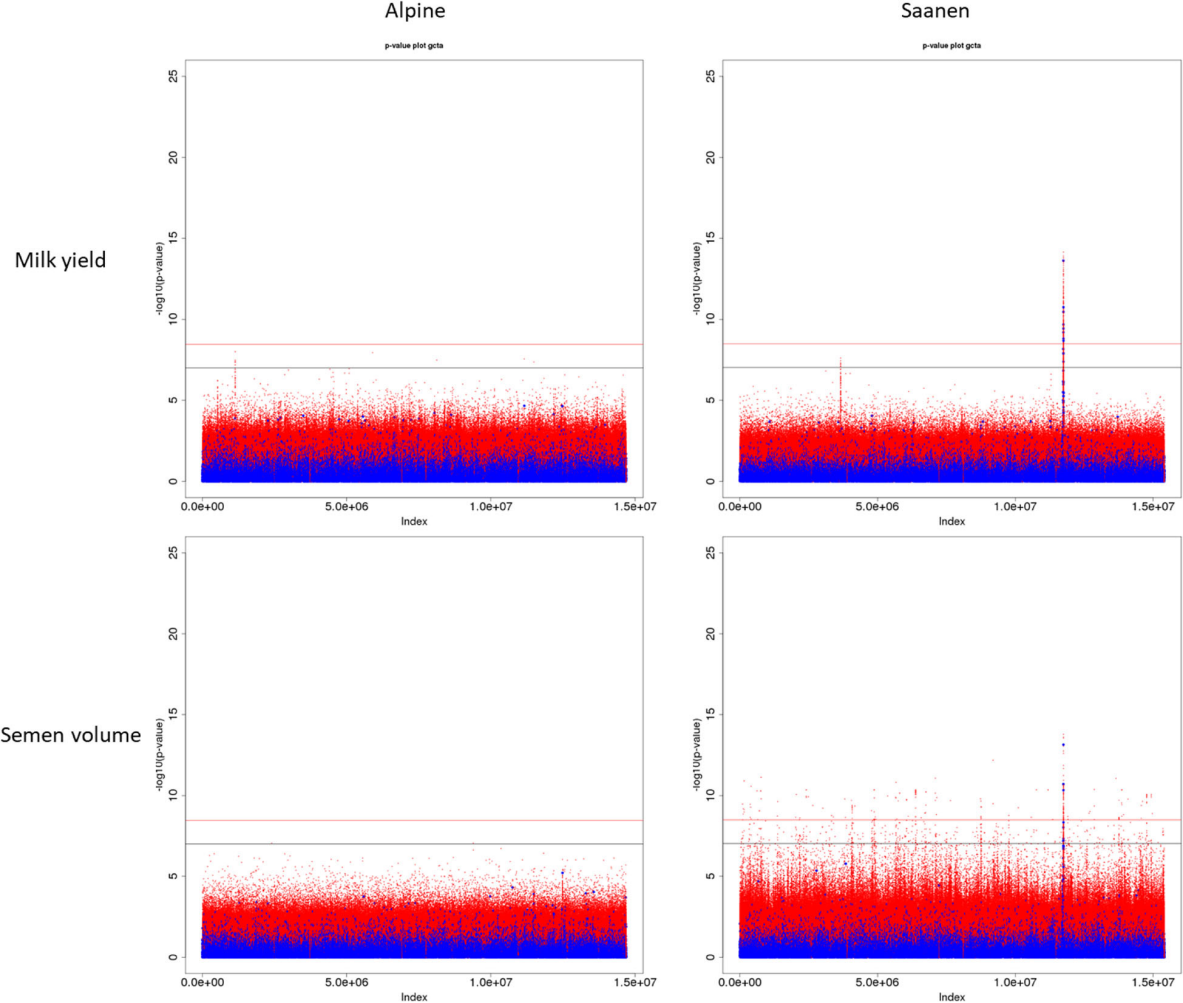
Manhattan plots for milk yield and semen production after multi-breed imputation of sequences using a French dairy goats panel. Chromosome significance threshold in black; genome-wide significance threshold in red. GoatSNP50 BeadChip variants in blue; sequence variants in red

The improvements provided by the imputed sequences can be easily assessed as 50 k markers genotypes (40,491 SNPs found in both the 50 k and sequence data) were directly replaced in sequence data using information of 50 k genotypes. They, therefore, underwent the same analysis using the same model, method and phenotypes. The significance levels tended to be higher with sequence data for all traits in the QTL regions (Fig. 7). Indeed, in the Alpine breed, sequence variants were systematically more significant than 50 k SNPs. In the Saanen breed, sequences variants were more significant than 50 k genotypes in every situation except for the semen volume trait when using a multi-breed reference panel for imputation. The sequence data also gave more refined peaks and a higher number of significant variants (Table 4).

#### Close linkage versus Pleiotropism test

As strong signals were detected for both semen volume and milk yield in the same region of chromosome 19. A Close Linkage versus Pleiotropism (CLIP) test as developed by David et al. [27] was implemented. We applied the CLIP test to sequence data imputed within-breed to determine whether the region is truly pleiotropic or if QTLs are physically close. We extracted 32,029 imputed sequence variants of chromosome 19 between 23 and 28 Mb to perform the analysis. The same analysis was performed on 50 k genotypes of the French Saanen breed and the test was detailed by Martin et al. [6]. The test compares two traits X_1_ and X_2_ and rejects the pleiotropy if the squared correlation between a combination of effects at the variant level (${\rho}_{X_1{X}_2}^2$), is below the minimal value it can take under the pleiotropy assumption multiplied by a factor *K*_∝_. *K*_∝_ is the αth percentile of the distribution of the ration of the square of the observed correlation to its minimal value under the pleiotropy assumption.

$$ {\rho}_{X_1{X}_2}^2<{K}_{\propto}\left[\sqrt{\left(1-\frac{1}{2N}\frac{\sigma_{y_1}^2}{\sigma_{X_2}^2}\right)}-\frac{1}{2N}\ \frac{\sigma_{y_1}^2{\sigma}_{y_2}^2}{\sigma_{X_1}^2{\sigma}_{X_2}^2}\right] $$

Where N is the number of animals included in the analysis, ${\sigma}_{X_1}^2$ and ${\sigma}_{X_2}^2$ are the observed variance of the traits and ${\sigma}_{y_1}^2$ and ${\sigma}_{y_2}^2$ are the variance of raw data.

## Discussion

### Imputation quality

In our study, we obtained similar imputation results with both a within-breed reference panel and a multi-breed sequence reference panel, provided that the breeds included in the reference population are quite similar to the imputed breeds (Fig. 2). We assume that using a wider reference panel covers best the genetic variability of the breed than the very little number of sequenced individuals of the breed in the VarGoats project.

Removing pedigree information before imputation strongly deteriorated the correlations (R), which decreased by 5.1 to 5.4% depending on the breed. CRs were less impacted, showing a decrease of 0.9 to 1.5% depending on the breed. In our conditions, a complete pedigree therefore seems useful to improve the accuracy of the imputed genotypes.

Even though the CRs obtained in this study were similar to those involving equivalent reference population sizes in other species, the correlations were significantly lower than in dairy cattle [28] or poultry [29]. Indeed, CRs ranged from 0.75 to 0.85 in Li et al. [28] and the genotype CR was around 0.8, depending on the chromosome, in Ye et al. [29]. However, the squared correlations for cattle breeds with similar population sizes ranged from 0.63 to 0.76 in Li et al. [28]. Binsbergen et al. [30] obtained results similar to ours with a reference population size of nearly 46 Holstein individuals. They performed direct imputation from 50 k genotypes to sequence level and obtained a mean correlation of 0.37 between true and imputed sequences.

One reason explaining our low correlations could be a negative effect of the large number of variants with a low MAF in our dataset (Fig. 4), for which the correlations decrease rapidly at lower MAF values (Fig. 3). Also, a slight drop in R was observed for variants with a MAF of 0.50 in Saanen goats (Fig. 3). This could be linked to the small number of variants with a high MAF (Fig. 4), and thus implies that an imputation error would have a major impact on the final correlation. Nonetheless, the correlations that we obtained even for high MAF were low in comparison with other studies in livestock [3, 29, 30] or humans [31] where the correlation between true and imputed genotypes could reach 0.8. As our imputation study involved very few sequenced individuals (33 and 40), a single imputation error would drastically reduce this correlation.

Another possible explanation is the considerable genetic diversity of the *Capra hircus* species [1, 32]. According to the French Varume project, the number of ancestors contributing to 50% of the gene pool is higher in French Alpine and Saanen (16 and 15 respectively) than in French dairy cow breeds: 7 in Montbéliarde and Holstein, 8 in Normande (Danchin-Burge, Institut de l’Elevage, personal communication). Besides, as shown by Carillier et al. [32], the LD is lower in French goats than in dairy cattle. A low LD might make phasing more difficult as the distance to a 50 k marker and therefore the number of potential recombinations increase, leading to imputation errors.

Moreover, the reference populations used for imputation were small and some individuals had initial low-depth sequences (coverage <10X). Parts of their sequence genotypes remain uncertain.

Nonetheless, most of the QTL regions previously identified with GoatSNP50 BeadChip data were detected on the sequence data with refined signals and increased significance, which suggests that the imputed sequence could be suitable for association analysis.

Nevertheless, the significance of the detected regions or the identification of new regions could be further increased by improving imputation quality. As only a limited number of animals in each breed were sequenced, some genotyping errors might be erased by increasing this number. According to Druet et al. [14], at a given sequencing effort, it is preferable to sequence more individuals at a lower coverage to detect rare variants and to call genotypes accurately than to sequence deeply few individuals.

### Association analysis

The *p*-values for sequence data were slightly higher than for 50 k data and clear distinct signals were identified when imputed sequence data were used for association analyses (Fig. 7). Sanchez et al. [10], conducted similar studies on the Montbéliarde dairy cattle breed and reported a major increase in the significance of the detected signals when using sequence data, rather than HD or 50 k genotypes. However, the imputation accuracy was greater than in our study. Two-step imputation has proven to be more efficient and in our case could dramatically increase the imputation quality. Binsbergen et al. [30] obtained a correlation similar to ours (0.37) when imputing directly from 50 k to sequence level using 46 sequenced individuals. This correlation increased and reached 0.65 when an intermediate HD step was introduced. A HD chip is not yet available for caprine species, it would be a very powerful tool that would improve imputation to sequence level by overcoming imputation errors.

Our results varied depending on the imputation scenario used to impute the available 50 k genotypes to sequence level. In the Alpine breed, using a multi-breed reference panel resulted in the detection of a new signal for milk yield on chromosome 2 (Fig. 5). This signal does not appear when using the 50 k genotypes (Fig. 7). Significant variants in this region are annotated for CRYBA2, CDK5R2 and FEV genes which are not explicitly related to the mammary gland or any metabolic path linked to milk production. According to the RumimiR database, the region close to the signal is rich in miRNA: 12 miRNAs are located in a range of 1 Mb around the signal in the caprine species. However, among them, only 2 are expressed in mammary tissue: chr2_2187 (29.47 Mb) and chr2_2972 (29.80 Mb) and 4 are expressed in the ovaries: _Novel: bta-miR-153 (28.57 Mb), _Novel: bta-miR-26a (29.39 Mb), LO-m0073 (29.80 Mb) and FO-m0047 (29.80 Mb). Their exact role and impact on milk yield is still unknown. Further analysis is therefore required to confirm or disprove the signal detected on chromosome 2 for milk yield.

In the Saanen breed, the multi-breed reference panel led to the detection of a new signal for milk yield on chromosome 5 while confirming the involvement of a large area on chromosome 19. Significant variants on chromosome 5 are annotated for MDM1 gene, however the link between this gene and milk production is not clear. According to the RumimiR database, there are a few miRNAs in goats that are also located on chromosome 5 near our signal: novel_mir299 (44.53 Mb) discovered in blood samples, chr5_4536_mature (42.17 Mb) and chr5_4548_mature (45.82 Mb). The two latter are expressed at high levels in mammary tissue but are located further away from the signal and their exact involvement in milk yield is not known. As the signal only appeared when imputing using a French multi-breed reference panel, PCA was performed using PLINK for the region of chromosome 5 between 44.804 Mb and 44.816 Mb to try to understand where the imputed frequencies in the region came from. No significant breed group was found using PCA which implies that the QTL might actually be present in other French goat breeds.

In Saanen goats, a larger number of significant variants for milk production were detected on chromosome 19 and deeper investigation is required in this area that is also linked to udder health and conformation [6] and semen production. Multi-breed imputation gave the highest number of significant variants for milk yield (Table 4). The variants are annotated for 91 genes including 3 miRNAs (mir195, mir324 and mir497). Top 10 most significant variants (*p*-values between 2.96 ∗ 10^−14^ and 1.09 ∗ 10^−15^) are annotated for 2 genes (SCIMP and ZNF232). One of the 10 most significant variants (26,099,146) is situated in an intron of an unknown gene (GENE_id401516). Our study does not allow us to isolate functional candidate genes with certainty as it would require functional analysis. Nevertheless, the proximity of our signal to the ALOX genes cluster constitutes an interesting lead as the latter genes are implicated in lipid metabolism.

For semen production and more particularly semen volume, using a multi-breed reference panel considerably increased the noise observed on Manhattan plots (Fig. 6) making it difficult to distinguish true signals from what could be false positives. DYDs for this trait are more precise as they are derived from multiple repeated data from on average 100 daughters per buck whereas semen traits are the bucks’ own limited number of repeated performances. The significance of the signal (Fig. 6) and the number of significant variants (Table 4) decreased slightly when a multi-breed reference panel was used compared with within-breed imputation. When imputing within-breed, 209 variants reached the chromosome significance level on chromosome 19. These variants are annotated for 61 genes. Four of the identified genes, (PELP1, ELP5, NEURL4 and CNTROB) are broadly expressed in testes. One gene (CHD3) is ubiquitous in the prostate, and another, YBOX2 (Y-box Binding Protein 2) is restrictedly expressed in testes. YBX2 is a member of the Y-box gene family that encodes a transcription factor and is specifically expressed in germ cells. Knock-out mice for this gene are of normal appearance but are sterile [33]. Mutations in this gene in humans are associated with male fertility disorders such as azoospermia and oligospermia [34]. A significant 23-bp deletion at position 26,614,373 was found in the French Saanen breed, close to the mature miRNA chi-miR-497 (26,614,406 – 26,614,427). The same variant is also located near chi-miR-195 (26,614,085 – 26,614,104). Both miRNAs are ubiquitously expressed in testicular cells and might have an impact on semen production traits.

A pleiotropic region for milk, type traits and udder health was previously identified on chromosome 19 for the Saanen breed by Martin et al. [6]. Our study confirmed that a 3.5 Mb region was involved in milk production. For milk yield and semen volume, when sequences were imputed within-breed, top 10 variants had MAF comprised between 0.39 and 0.44 in the Saanen breed. The CLIP test rejected the pleiotropy assumption. The observed correlation was estimated at 0.013 and the threshold not to reject pleiotropy was above 0.15. The two traits might therefore be controlled by two different mutations situated close to each other. Moreover, none of the top 10 variants is shared between the two traits. According to the estimated effects, the allele with the highest frequency in the QTL region decreases both SV (− 0.09 SD) and milk yield (− 0.51 SD). Such an association therefore shows a favorable condition for improving both semen quantity and milk production.

## Conclusions

This study provides insights on how to implement a robust quality check and an imputation pipeline based on caprine sequence data that will ensure the quality of subsequent analyses. New signals for milk yield traits were detected in both Alpine and Saanen breeds. Signals for semen and milk production traits were detected in the Saanen breed on chromosome 19. The latter regions however require further investigation and annotation to determine the genes involved and determine more precisely their impact. Imputation using a within-breed scenario appears to be more efficient because it is less time consuming. Signals detected after within-breed imputation show less noise and are more significant. However, due to the small size of our sequenced panel, within-breed imputation might not be able to detect smaller weaker signals. Increasing the number of sequenced animals should therefore be considered. Densifying the current genotyping array in the identified regions could corroborate their involvement in functional and production traits while removing potential imputation errors. In the same way, developing a HD chip for *Capra* species would improve the quality of imputation to sequence level by proceeding in two steps. Furthermore, functional analyses are required to confirm the involvement of identified genes in the studied phenotypes.

## Data Availability

The final sequence dataset will be made publicly available by the VarGoats Consortium. The use of the sequence data is under a data sharing agreement which is available here: http://www.goatgenome.org/vargoats_agreement.html and states that everyone will contact the VarGoats steering committee to discuss any publication plans that utilize this data to avoid the overlap of any planned analyses. Performance data and 50 k genotypes are not publicly available as they involve private professional partnerships.

## Abbreviations

AI: Artificial Insemination
ALOX: Arachidonate LipOXygenase
CDK5R2: Cyclin Dependent Kinase 5 Regulatory subunit 2
CHD3: Chromodomain Helicase DNA binding protein 3
CLIP test: Close LInkage versus Pleiotropy test
CNTROB: CeNTRoBin, Centriole Duplication and spindle assembly protein
CR: Concordance Rate
CRYBA2: Crystallin beta 2
DP: Depth
DYD: Daughter Yield Deviation
ELP5: Elongation acetyltransferase complex subunit 5
FEV: FEV transcription factor
GQ: Genotype Quality
GWAS: Genome Wide Association Study
HD: High Density
Indel: Insertion/Deletion
MAF: Minor Allele Frequency
MDM1: MDM1 nuclear protein
MiRNA: MicroRNA
MY: Milk Yield
NEURL4: Neuralized E3 ubiquitin protein ligase 4
PCA: Principal Component Analysis
PELP1: Proline, glutamate and leucine rich protein 1
QTL: Quantitative Trait Loci
R: Pearson Correlation
SC: Semen Concentration
SCIMP: SLP adaptor and CSK interacting membrane protein
SN: Spermatozoa Number
SNP: Single Nucleotide Polymorphism
SV: Semen Volume
WGS: Whole Genome Sequencing
YBOX2: Y-box Binding Protein 2
YD: Yield Deviation
ZNF232: Zinc Finger Protein 232
